# Pancreatic Emulsion in Wasting Diseases of Children

**Published:** 1873-05

**Authors:** 


### On the use of Pancreatic Emulsion in the Wasting Diseases of Children.

Horace Dobell, M. D. (*Practitioner*, October, 1872), proposes this remedy for that wretched form of atrophy, debility and marasmus in children, where every part of the body wastes away except the abdomen, the state described by Dr. Druitt in his *Vade Mecum* in the following few and graphic words: "Emaciation and voracity; the belly swelled and hard; the skin dry and harsh; the eyes red; the tongue strawberry colored; the breath fœtid; the stools dark-colored and offensive; the bowels sometimes costive, sometimes extremely relaxed; the patient usually dies hectic." The author desires to bring prominently forward that this state, provided there is no advanced lung disease, is rapidly cured by pancreatic emulsion given given in doses of a teaspoonful every four hours, and regularly persisted in until fat and flesh are restored. It is, of course, necessary that a proper diet should be insisted upon at the same time; but proper diet without the pancreatic emulsion will not do. In addition to the stress laid upon the influence of the salivary and pancreatic juices upon the digestion of starch, in Dr. Prospero Sonsino's paper, the author says we must not forget the action of the pancreatic juice upon fat; and it is probable that the two functions of the pancreas are sufficiently independent of each other that they may act separately. As shown by experiments, in addition to the action of the pancreas upon fats, it has the power to convert starch into glucin by simple mixture, and this property remains to a certain extent after the pancreas has exhausted its power of acting upon fat. It is possible, therefore, that in different states of depraved health, the one or the other of these properties may be deficient. It is evident that when the power of digesting fat fails to be developed at the proper time, the effect must tell with double force upon children already suffering from deficient digestion of starch.

The children who become the subject of the kind of wasting now spoken of are especially: (1) those suckled by mothers whose milk, though abundant, is extremely deficient in nutritive properties; (2) those brought up by hand; (3) those who, at a later period of childhood, have been subjected to similar chronic defects of diet. It is especially when the mother's milk is poor in fat and lactin that the child becomes dissatisfied and craving; and, in the majority of cases, it is this that first leads to the introduction of farinaceous food, under the popular nursery belief that it is satisfying. As Dr. Sonsino says, if this is given before the power of digesting starch becomes established, of course nothing but mischief can be the result. In the same way that the mother is deprived of fat elements by lactation, so is the child deprived of them by persistency in a diet deficient in milk. The injury is a double one, first by cutting off the supply of fat elements necessary for the protection of the tissues, secondly, by paralyzing the functions of the pancreas by prolonged inactivity. This latter is a point, the author thinks, deserving great attention, and thus accounts in great measure for the impossibility of restoring those ill nourished, wasted children by any kind of natural diet after they have been allowed to remain in a chronic state of defective nutrition. The author cites three of the very numerous cases where he has seen pancreatic emulsion administered followed by almost magical recoveries. No amount of milk or cream will take the place of the emulsion, the explanation why, notwithstanding milk is also an emulsion of fat, the author thinks turns upon the following points: (1) The fineness of the particles of fat in the pancreatic emulsion; (2) the permanent character of the molecular mixture; (3) the fact that different fats in the pancreatic emulsion, consisting, principally, of stearine, margarine and palmatine, have a high melting point, thus differing from the fat of milk, oleine, which has a low melting point.—*Boston Medical and Surgical Journal.*

